# Expert views on the factors enabling good end of life care for people with dementia: a qualitative study

**DOI:** 10.1186/s12904-015-0028-9

**Published:** 2015-07-25

**Authors:** Richard Philip Lee, Claire Bamford, Catherine Exley, Louise Robinson

**Affiliations:** Institute of Health & Society, Newcastle University, Baddiley-Clark Building, Richardson Road, Newcastle-upon-Tyne, NE2 4AX UK

**Keywords:** Dementia, End of life care, Experts, Qualitative, Palliative care

## Abstract

**Background:**

Dementia, of all long term illnesses, accounts for the greatest chronic disease burden, and the number of people with age-related diseases like dementia is predicted to double by 2040. People with advanced dementia experience similar symptoms to those dying with cancer yet professional carers find prognostication difficult and struggle to meet palliative care needs, with physical symptoms undetected and untreated. While elements of good practice in this area have been identified in theory, the factors which enable such good practice to be implemented in real world practice need to be better understood. The aim of this study was to determine expert views on the key factors influencing good practice in end of life care for people with dementia.

**Methods:**

Semi-structured telephone and face-to-face interviews with topic guide, verbatim transcription and thematic analysis. Interviews were conducted with experts in dementia care and/or palliative care in England (n = 30).

**Results:**

Four key factors influencing good practice in end of life care for people with dementia were identified from the expert interviews: leadership and management of care, integrating clinical expertise, continuity of care, and use of guidelines.

**Conclusions:**

The relationships between the four key factors are important. Leadership and management of care have implications for the successful implementation of guidelines, while the appropriate and timely use of clinical expertise could prevent hospitalisation and ensure continuity of care. A lack of integration across health and social care can undermine continuity of care. Further work is needed to understand how existing guidelines and tools contribute to good practice.

**Disclaimer:**

This article presents independent research funded by the National Institute for Health Research (NIHR) under its Programme Grants for Applied Research programme (Grant Reference Number RP-PG-0611-20005). The views expressed are those of the author(s) and not necessarily those of the NHS, the NIHR or the Department of Health.

## Background

Dementia, of all long term illnesses, accounts for the greatest chronic disease burden, with care costs in the United Kingdom (UK) estimated at £20 billion [1], and the number of people with dementia worldwide is predicted to double by 2040 [2, 3]. Currently one in three people aged over 60 years die with dementia [4]. People in the advanced stages of dementia experience similar symptoms to those dying with cancer [5] yet research shows professional carers find prognostication difficult [6, 7] and struggle to adequately meet their palliative care needs, with physical symptoms undetected and untreated [8, 9]. Thus the need to ensure good end of life care for people with dementia is a pressing issue.

A number of studies and reviews have identified key features of good end of life care for people with dementia [10–15]. Most recently a European study employed a Delphi approach, in which 89 experts defined optimal palliative care for people with dementia [14]; this iterative process resulted in 57 recommendations in 11 domains. The top five priorities identified in order of importance were: the optimal treatment of symptoms and providing comfort; person-centred care, communication & shared decision-making; family care and involvement; societal and ethical issues; avoiding overly aggressive treatment. However, an integrated review of evidence suggests that despite attempts to theoretically define what constitutes good end of life care, little work has been undertaken to understand how such care is supported or constrained in actual practice [10].

By way of context, in England in 2003 59.7 % of dementia-related deaths occurred in a care home, 36.0 % in hospital, 3.7 % at home and 0.3 % in a hospice or other palliative care institution [16]. The same study reports only 2.8 % of people in the Netherlands died in hospital, while 92.3 % died in a care home. In Belgium, there was a greater % of people dying at home (11.4 %) and a lower % dying in hospital (22.7 %) than in England. More recently the National End of Life Care Intelligence Network suggests that in England 39 % of people with dementia as an underlying cause of death died in hospital, 55 % in a care home, 4 % in their own home (with small numbers dying in a hospice or elsewhere) [17].

The provision of care in England (excluding hospital care) generally happens within residential care homes (often with minimal in-house clinical expertise and reliant upon community nurses and GPs), homes with nursing skills, and through care provision in people’s own homes provided by community nurses, GPs and/or specialist teams. The expertise of care staff and health professionals to deal with end of life care and dementia varies within and between settings and between localities.

## Methods

This study is part of a five year programme of research focused on supporting professionals in England to provide good quality end of life care to people with dementia. The aim of this phase of our study was to determine the views of experts in palliative and dementia care, through qualitative data collection, on factors which enable or constrain the delivery of good end of life care in dementia.

We conducted semi-structured qualitative interviews with 30 experts in dementia and/or palliative care in England. The interviews were conducted between October 2013 and March 2014. An initial list of ‘experts’ known for their national roles in policy making in the fields of dementia care and palliative care was developed by the broader project team in order to achieve differentiation by role (identified as academic, clinical, policy, business, not for profit) and area of expertise (dementia care, palliative care or both, Table 1). Sixteen participants came from this initial list. Sampling for the study was purposive and iterative and snowballing was used to identify additional participants. Potential participants were approached by email or telephone, provided with a summary of the research and given the opportunity to ask further questions. Of those contacted three did not respond and two declined; in both these cases the potential participants contacted colleagues who agreed to participate. Verbal consent was taken at the time of interview, including agreement for the interview to be audio-recorded.

**Table 1:** Role and area of expertise of participants

Role	Area of expertise		
	Dementia care	Palliative care	Dementia & palliative care
Academic	RESP3	RESP13	RESP1; RESP5; RESP7; RESP24
Clinician	RESP22; RESP29	RESP23	RESP6; RESP12
Academic/Clinician		RESP14	RESP9; RESP25; RESP30
Policy (including voluntary sector)		RESP15; RESP17	RESP8; RESP10; RESP16; RESP20
Policy/Clinician	RESP19		RESP21
Private residential care provider	RESP2		RESP18; RESP26
Training provider			RESP4; RESP11; RESP27; RESP28

A topic guide was used during the interviews to explore: established models of good practice in end of life care for people with dementia, specific examples of those models, components of good practice, differences and similarities between end of life care for people with dementia and other conditions, and the value of existing end of life care frameworks to dementia care. Participants were also given the opportunity to talk about other issues they deemed important to end of life care for people with dementia.

The interviews were transcribed verbatim and fieldnotes were made to aid analysis. A subset of transcripts were read and subject to open coding by RL and CB to identify issues discussed by the participants. The open codes were then used to aid thematic analysis [18, 19], which was conducted separately by RL and CB and collectively in two data workshops involving RL, CB, CE and LR. The subsequent coding frame was applied to further transcripts. Based on this analysis, a further analysis was undertaken focusing on the factors which contribute to the conduct of good practice in end of life care for people with dementia. It is this secondary analysis which we focus on in this paper and describe more fully in the results section.

The research was approved by Newcastle University Faculty of Medical Sciences Ethics Committee (00665/2013). The study is part of a five year programme grant funded by the National Institute of Health Research in England (NIHR PGfAR - RP-PG-0611-20005).

## Results and discussion

Thirty experts in dementia and/or palliative care were interviewed. From our analysis of elements of good practice discussed by experts it became clear that knowing the person with dementia, interpreting non-verbal communication, listening to and respecting family carers and ensuring personal and physical comfort, were recognised as important.

Knowing the person with dementia involved understanding their wishes and preferences and judging how and when to talk about dying with them. Knowing the person could be supported by ensuring continuity of care, a factor discussed in more detail below. Being able to interpret non-verbal communication was also viewed as an essential element of good practice, involving recognising the meaning of facial expressions, the meaning of sounds and realising that the signs of agitation may have underlying causes. Listening to and respecting family carers was discussed in terms of building on the existing knowledge of family members (including contributing to the interpretation of communication and providing insights in to a person’s life history), supporting existing relationships, providing post-bereavement support and managing the transition to a sharing of care with staff. Ensuring personal and physical comfort involved addressing symptoms in a proportionate manner, identifying and treating pain, and that features of comfort (a familiar, calming environment, appropriate room temperatures, and a knowledge of what comfort looks like for a particular person) are prioritised.

While this analysis contributed to our understanding of the elements of good practice, respondents also discussed the factors which enabled good practice to happen. It was apparent that these factors have significant implications for the performance of good practice; this became the focus of further analysis. The aim was to produce a focused account of the organisational factors experts considered crucial to good practice. Based on this additional analysis, we identified four related factors with important implications for good in practice in end of life care for people with dementia:

- leadership and management of care
- integrating clinical expertise
- continuity of care
- the use of guidelines and care pathways

Each is described in detail below, drawing upon data from the expert interviews.

### Leadership and management of care

There was much discussion by respondents of the importance of leadership, the responsibilities of the care leader and the management of care. Owners and managers of care homes were regarded as having a responsibility to resource care appropriately:

I think we also need to think about, particularly if we’re looking at care settings, the kind of management and ownership, what are their responsibilities…we could put a lot onto care home frontline care staff, they are working in a bigger system that may not resource them to be able to do that…guidelines or commissioning guidelines also need to make some statements around what resources need to be present in order for this to be done properly. Because otherwise what happens is the care staff have to do more and they do everything a bit less well. [RESP7, Academic]

For one interviewee, owners and managers of care homes contribute to valuing staff by understanding the demands of their work and anticipating problems:

…one care home will be brilliant and another care home would be disastrous and that’s about leadership of the care home and it’s about valuing the staff. If the staff are not valued they feel very taxed about caring for the psychological needs and behavioural needs of people with advanced dementia because, it’s tough and if they’re not valued for the work that they do and management is aloof and doesn’t understand the difficulties and the shortage of staff, there will be difficulties and it won’t be a nice place to be cared for. [RESP30, Academic/Clinician]

While interviewees noted that owners and senior managers should be attuned to the work and concerns of staff, they also felt that managers should also take a role in utilising their staff appropriately for end of life care:

a manager…he put his best staff on where the person needed end of life…he chose the staff who had the best interpersonal skills and the best knowledge, so he said that if you had, if somebody was needing end of life care they would get the best, that’s awful, but when he was allocating his staff, they tend to get the best staff, because they thought it was more important that they were expert than perhaps going to somebody who, I don’t know, somebody with a physical disability who could say exactly what they wanted. [RESP2, Private residential care provider]

Managers were also viewed as having an important role in demonstrating good practice to other members of staff:

good leadership, generally, or the better leadership is generally where you see the General Manager out on the floor modelling. Not all the time, but they’re there, they know the family members, they know the staff members, not just know them by name but they actually know their life stories and what’s really important to them, and they share theirs. [RESP26, Private residential care provider]

However multiple levels of management and corporate policies within larger residential care companies were recognised by one expert as a possible constraint on local service development:

But some of those bigger ones struggle because of that. Too many tiers of management often. So you might have a manager in the home who really wants to do this [end of life care training], but the regional manager is the sticking point. [RESP27, Training Provider]

Whilst highly skilled and well supported care staff were viewed as integral to good practice, experts also explicitly commented that low levels of pay and investment in the development of care staff were barriers to achieving this in practice:

…how much money people want to spend on care homes is fiercely screwed down. People who are providing the care are usually very poorly paid. Many of them are very keen to do things properly. The staffing levels may not be great and the knowledge may not be great. [RESP9, Academic/Clinician]

care homes inhabit a low wage economy, they are, unfortunately, probably losing a quarter of their staff every 12 months, and in the main they’re underqualified, in many settings still they’re untrained. [RESP18, Private Residential Care Provider]

In summary, despite a number of barriers within care homes settings such as low wages for care staff, high staff turnover and inappropriate numbers of qualified staff, most experts recognised that leadership and management in care homes, with staff support and working examples of good practice, was crucial for good quality care. This area of practice however needed to be complemented by the appropriate integration of clinical expertise in palliative care.

### Integrating clinical expertise in palliative care into routine care settings

Rather than simply extending existing palliative specialisation into dementia care, one interviewee suggested that palliative expertise needs to be more appropriately and widely distributed amongst generalists and specialists:

I’m not sure that we actually need a specialist palliative care in the way that we have specialist palliative care in cancer care. I think we need a more distributed model, so that, for example, psychiatrists for older people and community psychiatric nurses have some of the those specialist skills in palliative care, and that they can employ them in the community, rather than waiting to refer on to some sort of specialist group. [RESP13, Academic]

Another suggested a similar approach, but voiced concern about the understanding of dementia among palliative care specialists:

…getting the generalists to work together and the specialist acts as an advisor…they’re a backup rather than a kind of main way of taking it forward. I think that’s the kind of thing to think about. I don’t think palliative care really understands dementia, to be honest. [RESP7, Academic]

The distribution of palliative care skills should, for another respondent, occur to the extent that all staff are aware:

…rather than it being a specialist thing, the skills of palliative care are there for everybody. So, if you have dementia and you’re dying, in a nursing home, for example, and…you may not be seen by a palliative care team, are there things that…any member of staff should be aware about palliative care. [RESP19, Policy/Clinician]

For these respondents the desire for a distribution of expertise is a response to concerns over the timely implementation of end of life care and the limited capacity of specialist services. The first recognises the role of professionals with on-going contact with people with dementia (old age psychiatrists and community psychiatric nurses), while the second suggest that the skills of palliative care should be developed by generalists working closely with people with dementia, with advice from specialists. The third draws attention for the need for all care staff to have some awareness of palliative care.

The up-skilling of generalist clinicians was seen by some interviewees as the right approach to integrating such expertise, with the more typical situation comprising routine care provided by a GP especially in patients with ‘uncomplicated’ dementia:

…the GP providing the normal care for the person. I think that for a lot of people with dementia, they don’t actually need anything more than that, in the sense that they haven’t got any difficult behaviours, they haven’t got depression, they haven’t got delirium, they’re not in terrible pain, so they don’t need that specialist palliative care input. They need very basic terminal care and it’s completely within the remit of general practice to provide that. The only caveat, which I suppose is a caveat for all of these things, is you’re then relying on the quality of the people providing the service. [RESP25, Academic/Clinician]

However this expert also recognised that integrating palliative care expertise into the usual care team, for example where a specialist nurse in palliative care visits a care home, can help to ensure people with dementia enjoy continuity of care:

…a [palliative] nurse going into care homes [is] a very good model for that end bit of palliative care because then, you’ve got somebody who really does have specialist knowledge but you’re also trying to keep people where they are as well. [RESP25, Academic/Clinician]

Experts said that clinical and palliative expertise has an important role to play in end of life care for people with dementia, but this needs to be responsive to the co-morbidities of the people with dementia:

people who are, are dying with their dementia and only their dementia…so the kind of not very difficult dying, and have dementia, and then those that have really quite difficult symptom management issues related to something else, such as motor neurone disease, cancer, and have dementia…So they might be quite distinct, I think. [RESP23, Clinician]

While this expert recognised palliative expertise should be appropriate to the clinical situation, another interviewee suggested that palliative care had a greater role to play in managing some of the physical causes of distress:

I think specialist palliative care need to really look at how they can support the wider, non-cancer community. Looking at symptom management, how do you manage pain control while in dementia, a number of other areas… [RESP8, Policy]

The issue of limited capacity was discussed by another interviewee:

specialist palliative care services are not confident in this and therefore do not go out of their way to seek referral for people with advanced dementia, partly to do with confidence in looking after people, partly to do with, I think, to do with fears of being swamped etc. and also because locally there are often good services for dementia, or at least, perceived to be good services for dementia. The other side of that coin, of course, is that people with dementia are not often referred to palliative care services. [RESP16, Policy]

The integration of clinical expertise was viewed as contributing to the pursuit of personal and physical comfort for people with dementia at end of life, if done sensitively. Generalists, informed by this kind of expertise, might be better placed to administer care through their existing knowledge of the person with dementia and relationship with family members. Continuity of care (discussed below) can contribute the effectiveness of clinical expertise, with the integration of expertise assisting on-going care work.

### Ensuring continuity of care

One of main advantages of having continuity of care is that staff can develop their knowledge of a particular person (including routines, biography, preferences and relationships), ideally before the onset of advanced dementia makes verbal communication difficult. The development of this knowledge relies upon continuity of staff, in particular a key individual:

having somebody there for the person with dementia and their carer, who is a known person who they can, can help them towards their care, navigate them through the system, whatever you want to call them, that key worker role is really important…so a relationship can be built…[RESP10, Policy]

that person acting as that sort of key worker. So providing that continuity of care for the person in their family I think is particularly important for somebody with dementia and end of life care needs. [RESP21, Policy/Clinician]

There was also recognition that it may be unavoidable that a person with dementia will move between different key professionals:

it probably won’t be the same person all the way through, ‘cause the baton in continuity will move on, but it may be the memory clinic people at the start, it may be the GP in the middle, and it may be the care home more towards the end. [RESP23, Clinician]

The co-ordination of a person’s care could also impact on continuity, recognised by one respondent who suggests a lead care manager or service is required to organise care in advance of a crisis event:

there’s no co-ordination and I think there may be some success to be had is where there is an individual, or an individual service, is actually co-ordinating the pathways for people with dementia because it’s usually crisis driven, it’s reacting to crisis, and depending on where that person is in the journey as to which service eventually takes up the challenge and looks after them, but there’s no, as far as I’m aware there are no consistent approaches or no approaches that are fairly robust in their approach. [RESP6, Clinician]

For another interviewee, care should follow the person with dementia and their family (despite the problems associated with funding models):

I think there has been a huge move towards trying to have community based palliative care…have care follow the patient, so community based visits, intensive home support, models where you pay an awful lot of attention to carer support as well because, while not everybody with dementia will have a family carer, many will and many will be supported by another older person…I think the trouble in England is that, we’re really being held back by funding models, so the development of these is not as refined as it should be. [RESP13, Academic]

Although residential settings can offer a level of continuity of care (subject to staff turnover), some interviewees highlighted the implications of management and staff not being able to recognise dementia or deal with advanced dementia and dying:

if the care homes could identify better with the help of obviously, clinicians, who has dementia that just raises the ante if you like, on how much knowledge they really ought to have about how to manage people with dementia – that’s not just really end of life but it’s the whole…from admission to care home, through to end of life and I think, you know, those care homes where they’ve really been proactive in getting to know dementia and training up the staff and so forth, have seen vast improvements in their quality of care and I think that includes end of life as well. [RESP29, Clinician]

staff actually talk among themselves and they kind of have a little bit of gut instinct and they kind of say ‘oh you know we think that this person is dying’, but actually there’s a general reluctance to make that explicit and formalised and the other kind of thing is that diagnosing dying is often left to medical professionals and yet they are the one, like GP who’d probably only visit once a week or something into a care home, they are often the people who don’t know the patient well or the rest very well, but it’s within a team, be brave enough to recognise that actually somebody is potentially dying [RESP5, Academic]

Being able to respond to dying and death within a care setting contributes to the continuity of care, often through a reduction in unnecessary hospitalisation. Not being too quick to act upon a change in condition was seen as a potentially positive response if that meant unnecessary hospitalisation could be avoided:

care staff want to do something…the biggest benefit for the patient is not to send them to hospital, that that will only cause harm. To have the confidence to see that in a very positive reframe, if you like, “That I’m really doing something really important here, by keeping them here” I think is fundamental. [RESP23, Clinician]

people with dementia at the end of life often get wheeled into hospital in their last few days, simply because sometimes the skills or the confidence of the staff in care homes isn’t there. [RESP20, Policy]

In these extracts the connection between developing competency and ensuring continuity of care is apparent. Where hospital admission does happen, retaining an element of continuity was seen as important:

Can we, without intruding, send [care] staff into the hospital with the person, you know? And how would that be paid? And it might seem like a cost to them, but actually, that environment is not geared up towards dementia. We cared for this person, so actually, I’d see a little bit of investment, financially releasing some staff to accompany this person, may save money down the line. The person won’t be in hospital as long. We will be able to communicate more effectively. We’ll have somebody that will be with them that will help them eat and drink, you know, who knows their daily routine. [RESP17, Policy]

Continuity of care could ensure that those most involved in a person’s care at end of life have a knowledge of the person, that comfort is supported by familiar surroundings, and that staff understand verbal and non-verbal communication. Importantly, continuity of care means that existing relationships with family members are not disrupted.

The integration of clinical and specialist expertise, and the leadership and management of care are steered by the use of guidelines and frameworks for end of life care. The final theme we focus on is the use of guidelines in delivering good practice.

### The use of guidelines and care pathways

Guidelines and frameworks were viewed in a number of ways by experts interviewed. In England, improving end of life care has been a national policy priority for many years. There has been an extensive dissemination of ‘good palliative care practice’ guidance for example, the Gold Standards Framework (GSF) [20], and care pathways for the final days of life, such as the Liverpool Care Pathway for the Dying Patient (LCP). The LCP has recently been phased out of practice following an independent, national review [21] and this generated considerable debate in terms of its use and relevancy for people with advanced dementia. Given our respondents were based in England, the prevalence of discussion about the LCP was perhaps unsurprising. However, their thoughts on this issue are relevant to practitioners working in different health and social care contexts:

there’s been quite a lot of disappointment expressed in [region] that the Liverpool Care Pathway has been removed because it’s now much more… I mean, it’s obviously got to be patient centred, but guidelines can be quite helpful in steering people and make sure they haven’t forgotten aspects of end of life care, I think, [RESP29, Clinician]

So I’m a very, very strong critic of the Liverpool Care Pathway, and I was delighted when it was - you know, it was brought to a stop. [RESP14, Academic/Clinician]

The structure of Liverpool Care Pathway, the principles behind Liverpool Care Pathway were fine. It was the delivery that was lacking. In dementia care the delivery was really compromised by, as I’ve said, this unpredictability of when does life end? [RESP18, Private Residential Care Provider]

These three extracts reflect a diversity of opinion over the future of the LCP, moving from disappointment, to delight and then to a more cautious assessment which recognises the importance of implementing the principles in practice. The relationship between the design of LCP and its specific use in end of life care in dementia was the focus of much discussion, with experts recognising the potential value of the LCP in this area but voicing caution over its application and use in practice:

I think the Liverpool Care Pathway was perfectly relevant to dementia, but only in those last few days. So I think it was as relevant to dementia as it would be to any other condition [RESP22, Clinician]

…with the Liverpool Care Pathway there needs to be a lot of sensitivity and a lot of support, you can’t leave this to junior staff, which is often where the Liverpool Care Pathway has come unstuck. That’s not to say junior staff can’t be involved but they shouldn’t be, the people leading this, they should be helping to enact something that’s been agreed with people who have experience and expertise. So I think this has got to be led by more senior people. [RESP12, Clinician]

Despite the LCP being phased out, it has a legacy in local plans and approaches to end of life care:

…but actually the nursing staff were tending to do the sort of things on the Liverpool Care Pathway anyway, before anybody said to them, “This is the Liverpool Care Pathway.” [RESP25, Academic/Clinician]

I’ve come across a myriad of, of local end of life initiatives, sort of just been applied to people with, with dementia…they tend to be hybrids of GSF [Gold Standards Framework] and LCP. So, with GSF kicking in long before death is imminent and then following the recommendations of LCP. So I see them more as a variant on a theme. [RESP18, Private Residential Care Provider]

The first extract suggests that elements of the LCP were already embedded in good practice for end of life care. The second extract suggests that local, formalised approaches to end of life care have drawn heavily on the LCP and the Gold Standards Framework (GSF), the latter an approach to changing care practice in relation to end of life care [20, 22]. More than a set of guidelines, the GSF is viewed by some as a means of achieving organisational change in terms of end of life care, though its application to end of life care for people with dementia is still being worked through.

the Gold Standards Framework. It’s a well-used tool…it’s not a nationally accredited tool with something like, a body like the UK Accreditation Body. They do have a dementia model, and personally, where it’s been used in care homes, has brought about culture change and therefore better outcomes for people who use services, and for their carers and for the staff. So there is an improvement, and certainly, a lot of commissioners look for the Gold Standards Framework. [RESP17, Policy]

With respect to the UK GSF initiative, one interviewee with expertise in this area commented that while cost might be cited as reason not to undertake GSF, those tasked to facilitate the implementation of GSF by services can also impede service change by failing to involve staff:

…you get some people who aren’t actually facilitators, they – they’re not naturally like that… they’re doing everything and they – rather than empowering the care home staff, which is what this is all about, they disempower them. [RESP27, Training Provider]

Elements of this extract correspond to the first factor discussed in this results section; the successful implementation of guidelines and frameworks depends on leadership and management.

Discussion of guidelines and tools focused on the use and demise of the LCP, with some discussion of the GSF and local policies. The importance of the interpretation and implementation of guidelines for practice was emphasised by some interviewees, in particular how the use of guidance should be informed by knowing the person and caring for them with compassion.

## Conclusion

In this paper we have considered the factors which influence good practice in end of life care for people with dementia in England, based on the analysis of interviews with experts. Four key factors were identified: leadership and management of care, integrating clinical expertise in palliative care into routine care settings, continuity of care and the use of guidelines and pathways. The relationships between these factors are important. For example, leadership and management of care was discussed in terms of its importance for the successful implementation of guidelines, while the appropriate and timely use of clinical expertise could prevent hospitalisations and so ensure continuity of care. A key consequence of a lack of integration across health and social care is the undermining of continuity of care, as identified by a number of authors [12, 23, 24]. Experts interviewed regarded unnecessary hospitalisation as a disruption to continuity of care, and they supported people with dementia remaining in familiar environments and being cared for by people known to them. Hospital settings, despite offering specialist medical care, are not necessarily the most appropriate place of care for people with dementia at end of life [25].

The importance of the organisational processes to end of life care has been recognised in previous studies. In particular, the need to integrate services and expertise across social and health care has been highlighted [11, 26, 27]. It has been argued that problems with delivering good end of life care for people with dementia reflect a lack of co-ordination rather than a lack of expertise [26]. Care homes have been identified as services which would benefit from integration into other systems of end of life care in order to enhance care [11]. In a comparison of palliative care for people with dementia and cancer across 5 European countries, the authors discuss how working across health and social care systems is a common source of difficulty when providing end of life care for people with dementia [27]. The international evidence for adopting a palliative care approach in dementia is equivocal, largely due to a lack of high quality evidence [28, 29]. Specialist palliative care provision to people with advanced dementia is more common in the United States [30] and dementia ‘hospice’ units have been established [31–33]. However in the UK, access to specialist palliative care by families living with advanced dementia is limited [34, 35], often facilitated via local projects [36].

A key theme in our findings was the need for better integration of palliative care into dementia services. Previous research has also found a lack of effective working between specialist dementia services (old age psychiatry in particular) and palliative care teams as a barrier to delivering better quality care for people with advanced dementia [37, 38]. Specific reasons to explain this ‘disconnect’ between these two key specialist services have been identified [27, 39, 40], but examples of effective solutions being delivered in real world practice is limited. In the United States, a ‘dementia hospice’ has been shown to be successful [32, 33] while in the UK the creation of specialist community-based teams have been suggested [41, 42]. However, examples of such innovative, joined-up care remain limited and often geographically localised [36, 43]. The publication of a consensus White Paper defining optimal palliative care provision for people with dementia may provide a good practice framework for helping service providers develop more integrated models of care [14].

There are some limitations to our study. Our approach to the sampling of national and local experts was pragmatic and purposive and based on team meetings, snowball sampling and the identification of experts through searching. One consequence was that discussions focused primarily on end of life care in care home settings. Factors influencing end of life care in other settings may differ. In addition, we chose to identify experts only in England consistent with the focus of the broader study on improving practice in this country; international comparisons may have added to, and broadened, our understanding of the organisational issues identified. In addition, the views presented are those of experts; individuals who may no longer be directly involved in care delivery or service provision. Comparisons with data based on interviews and focus groups with service managers and frontline staff may further our understanding of the issues from different perspectives; we are undertaking data collection with these groups and will report on the implications for the current paper in future publications.

### Implications for future research, policy and practice

The results of our study reveal that national policy experts consider continuity of care and the integration of specialist palliative care expertise into routine care settings to be important factors influencing good quality end of life care for people with dementia. Interestingly, in England the latter has been the core philosophy of a national End of Life Care Strategy, introduced over a decade ago, with the appointment of national leaders and considerable resource invested in training both community and hospital staff in palliative care principles. Meanwhile, the Netherlands has achieved such a combination via a unique approach, the creation of a new medical speciality, the *nursing home physician*, who provides dedicated care for people with dementia, towards and at the end of life, specifically in care homes [44]. Further research is needed to explore how the views of those professionals directly responsible for providing and delivering such care compare with those of national policy makers who recommend it as best practice.

Frameworks such as the GSF can offer an alternative approach to changing practice, but service level implementation can be both enabled and constrained by leadership and existing guidelines and protocols. It is interesting to note the emphasis experts place on the role of guidelines and care pathways, especially in view of the recent controversy in England around the use of the Liverpool Care Pathway, a tool which is also widely used internationally. Following much criticism from families and a lack of high quality evidence to support the effectiveness of its use, findings from an independent committee have led to its removal from practice. In England, the publication of new guidance emphasising the local implementation of common principles rather than a specific pathway [45] is now recommended; how front line staff adapt in practice, without the help of a specific framework, and which end of life care guidelines they find useful in dementia where the dying trajectory is long and unpredictable, is another area in need of future research.

Although the opinions of experts are highly relevant, research which directly observes good quality care in actual practice is urgently required to inform the development of good practice guidelines and tools which are helpful and relevant to different clinical situations.

## Abbreviations

(GSF): Gold Standards Framework
(LCP): Liverpool Care Pathway for the Dying Patient
